# Effect of Antibiotics on Clinical and Laboratory Outcomes After Mandibular Third Molar Surgery: A Double-Blind Randomized Clinical Trial

**DOI:** 10.3390/antibiotics14020195

**Published:** 2025-02-13

**Authors:** Fatemeh Soleymani, José Eduardo Maté Sánchez de Val, Artiom Lijnev, Mehrdad Makiabadi, Carlos Pérez-Albacete Martínez

**Affiliations:** 1 Health Sciences PhD Program, UCAM-Universidad Católica San Antonio de Murcia, Campus de los Jerónimos nº135, Guadalupe, 30107 Murcia, Spain; 2 Department of Biomaterials, Engineering, Faculty of Health Sciences, UCAM-Universidad Católica San Antonio de Murcia, Guadalupe, 30107 Murcia, Spain; jemate@ucam.edu (J.E.M.S.d.V.); alijnev@ucam.edu (A.L.); cperezalbacete@ucam.edu (C.P.-A.M.); 3 Independent Researcher, 30007 Murcia, Spain; makkim891@gmail.com

**Keywords:** anti-bacterial agents, dry socket, infections, pain, trismus, antibiotic resistance, saliva, prostaglandins, third molar removal

## Abstract

**Objectives**: This double-blind, randomized clinical trial aimed to evaluate the impact of 2 g of pre-operative amoxicillin on postoperative clinical outcomes and salivary prostaglandin E2 (PGE2) concentration following mandibular third molar removal. **Methods**: Eighteen healthy adult patients requiring impacted mandibular third molar extraction were randomly assigned to two groups: an experimental group (EG) receiving 2 g of amoxicillin and a placebo group (PG) receiving empty capsules, one hour before the surgery and before taking the first saliva sample. Primary outcomes measured were pain levels at different time points and salivary PGE2 concentrations measured before, 24 h, and 7 days after the surgery, while secondary outcomes included changes in maximum mouth opening (MMO) immediately after the surgery at 1 day and a week post-surgery, and facial swelling at 24 h and 7 days post-surgery. **Results**: The results showed no significant differences between the EG and PG in terms of pain levels, salivary PGE2 concentration, MMO changes, or facial swelling at different time points (*p*-values > 0.05). One instance of surgical site infection was noted in the PG in the 7-day follow-up session, but it was not statistically significant (*p*-value = 0.303). Correlation analyses indicated that a higher number of sutures and a higher difficulty index of surgery were associated with increased pain, while longer surgery duration and osteotomy were linked to more MMO changes and facial swelling (*p*-values < 0.05). In addition, while longer surgery duration and performing tooth section were correlated with lower PGE2 concentrations, PGE2 concentrations were positively correlated with pain levels (*p*-values < 0.05). **Conclusions**: Based on the results of this study, administering 2 g of prophylactic amoxicillin did not significantly affect postoperative clinical or laboratory outcomes in healthy patients undergoing mandibular third molar surgery.

## 1. Introduction

The removal of third molars is frequently recommended for various pathologies, including pericoronitis, caries, bone loss, resorption of the second molar, or as part of orthodontic treatment [1]. Pain and swelling are common post-surgery and can impact patient recovery and quality of life [2]. In 2012, a model for post-surgery blood inflammatory changes was presented [3]. According to this model, white blood cell counts peak 24 h after surgery, with an acute increase in neutrophils and a decrease in lymphocytes persisting for a week. Additionally, C-reactive protein (CRP) and fibrinogen levels rise, peaking on the seventh day post-surgery and returning to baseline after three months [3,4,5].

Saliva has garnered significant attention due to its rich composition of detectable factors, including inflammatory markers such as prostaglandins, and the ease of obtaining saliva samples, owing to their non-invasive collection method and on-site applicability [6]. Research has explored salivary alpha-amylase, total protein, immunoglobulin A, and prostaglandin E2 (PGE2) levels after third molar surgery [4,7,8]. However, to the best of our knowledge, no previous study has investigated the effect of prophylactic antibiotic therapy on salivary PGE2 levels.

Surgical site infections and alveolitis are possible complications following third molar surgeries, for which dental practitioners often prescribe antibiotics. The incidence of infection after third molar surgery is 1–16% [9], while alveolar osteitis occurs in 0.5–5% of patients, post-extraction [10]. Although some review articles conclude that antibiotic prophylaxis significantly reduces the risk of alveolitis and surgical site infections after third molar extraction [11,12,13,14], other reviews dispute these findings [11,15]. However, a recent overview of reviews highlighted methodological concerns in systematic reviews, indicating that the overall confidence in their results was low to very low [16].

The excessive use of antibiotics in dentistry is increasingly worrisome due to the escalating threat of antibiotic resistance. Survey data reveal that antibiotics are included in 62% of prescriptions by Indian dentists and 72% of those by Croatian dentists [17]. According to the ADA, prophylactic antibiotics are generally not recommended for healthy patients prior to dental procedures [18]. However, in Spain, approximately 82.7% of dental clinicians tend to prescribe antibiotics following all third molar surgeries [19]. In addition to the long-term risk of antibiotic resistance, routine antibiotic prescriptions after dental surgeries could impose a significant financial burden on public health systems [20]. Furthermore, the efficacy of antibiotics in reducing complications such as swelling, trismus, and pain remains a topic of debate, with studies showing mixed results on their benefits when administered before or after surgery [15,21,22]

Given the controversies and the critical need to reduce unnecessary antibiotic prescriptions in dentistry [11], this double-blind randomized clinical trial aimed to evaluate the impact of pre-operative antibiotic therapy on postoperative consequences following mandibular impacted third molar surgery. The study was designed with two components: a clinical and a laboratory level. In clinical part, this study compares the objective and subjective clinical outcomes between the placebo and experimental groups, assessing the effect of a 2 g pre-operative dose of amoxicillin, and evaluates the correlation between clinical outcomes and basic/surgery-dependent variables. In the laboratory part, this article investigates the differences between the two arms of the study regarding the salivary prostaglandin E2 (PGE2). The primary hypothesis of this study is that antibiotic prophylaxis does not significantly affect pain levels or PGE2 concentrations in saliva after mandibular third molar extraction. The secondary hypothesis is that antibiotic prophylaxis does not significantly impact maximum mouth opening (MMO) or facial swelling post-surgery.

## 2. Results

**Figure 1:**
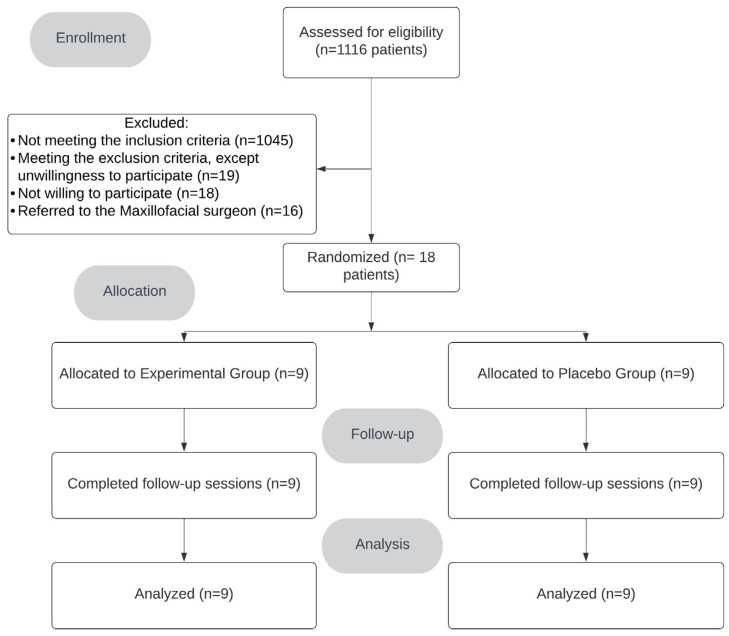
CONSORT diagram presenting the details of excluded and included participants.

Eighteen persons (nine in each group: five females and thirteen males) completed the clinical trial, with no dropouts after the study began (7-day follow-up) (Figure 1). All participants had a thick gingival biotype and BPE index < 2 in all teeth examined. The two groups were similar in their baseline data and surgery details, except for difficulty index (DI), which was significantly higher in the Placebo Group (PG) (*p*-value= 0.016) (Table 1). However, the mean surgery duration in each group was not significantly different (1605.33 ± 1478.58 s in Experimental Group (EG) and 1205.11 ± 964.15 s in PG, *p*-value = 0.506). In addition, no significant difference was found between the two groups regarding baseline PGE2 levels, confirming their similarity based on this variable (*p*-value = 0.246).

During the follow-up sessions, four participants in EG and five in PG received antibiotics due to severe pain and swelling (*p*-value = 0.629). However, since the second measurements were taken after 24 h and before the initiation of antibiotic treatment, these participants were not excluded from the study. Notably, there was no dry socket and one occurrence of surgical site infection in the PG, but it did not reach statistical significance (*p*-value = 0.303).

### 2.1. Pain

**Figure 2:**
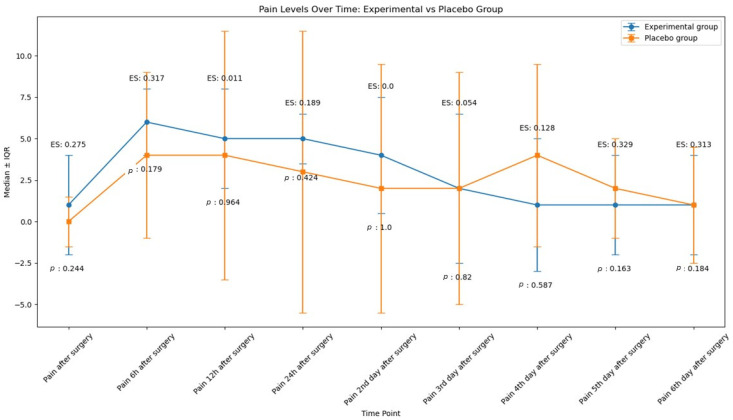
Average pain levels reported by participants in each group. (ES: Effect size, *p*: *p*-value).

Table 2 and Figure 2 show the pain levels reported by the EG’s and PG’s participants immediately after surgery up to six days post-surgery. Initially, both groups experienced a significant increase in pain within the first 6 h post-surgery, with EG reporting higher pain levels than the PG. From 6 h to 48 h post-surgery, pain levels remained significantly higher than immediately after surgery for both groups. After this period, both groups showed a gradual decline in pain levels over the following days. However, there was no statistically significant difference in pain reported between the two groups at any time point (*p*-values > 0.05, effect sizes ranging from 0.0 to 0.3). Factors such as gender, tooth sectioning, and osteotomy did not influence the reported pain levels (*p*-value > 0.05).

Table 3 displays the results of the correlation analysis between pain and various variables, which indicate a moderate, significant positive correlation between the number of sutures and pain reported at both 6 and 12 h post-surgery. Additionally, pain on the sixth day post-surgery showed a significant moderate positive correlation with DI and the performance of osteotomy. Interestingly, a moderate, significant negative correlation was observed between age and pain reported immediately after surgery. Conversely, no significant correlation was found between pain and other variables.

### 2.2. Salivary PGE2 Concentration

**Figure 3:**
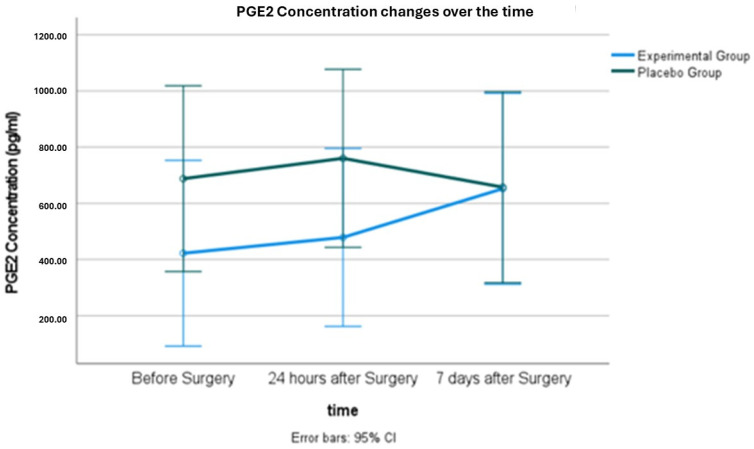
Mean Salivary PGE2 concentrations in the two groups at different time points.

Table 2 and Figure 3 present the mean salivary PGE2 concentration in each group and its changes over time. The repeated measures ANOVA test revealed no significant difference in the changes in PGE2 concentrations over time between the two groups (*p* = 0.244). An Independent Samples *t*-test was used to evaluate each time point separately, and the results are presented in Table 2.

Correlation analysis revealed that tooth sectioning was correlated with lower PGE2 levels in saliva one day post-surgery. More surgery time was moderately and significantly correlated with lower PGE2 levels after 7 days. No significant correlations were found between PGE2 changes after 24 h or 7 days and other variables.

### 2.3. Maximim Mouth Oppening (MMO) Changes

**Figure 4:**
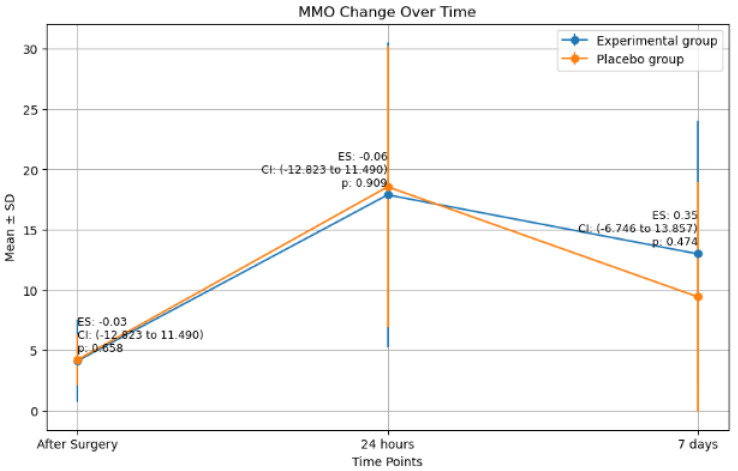
Mean MMO changes in each group at each time point. (ES: Effect size, CI: Confidence interval, *p*: *p*-value).

Patients exhibited reduced mouth opening 24 h post-surgery, which improved by the seventh day but did not return to pre-surgery levels (Figure 4). No significant differences in MMO changes were observed between the two groups at any time point, with *p*-values of 0.658, 0.909, and 0.474 for changes immediately, 24 h, and 7 days post-surgery, respectively, and effect sizes between −0.06 and 0.3.

Correlation analysis revealed a moderate, significant positive correlation between DI and MMO changes at 24 h, which shifted to a weak, non-significant positive correlation at 7 days. A significant, moderate positive correlation was found between the duration of surgery and MMO changes immediately after surgery, and at 24 h after surgery. Osteotomy showed a strong-to-moderate correlation with increased MMO changes at 24 h and 7 days. No correlation was found between MMO changes and other variables (Table 4).

### 2.4. Facial Swelling

**Figure 5:**
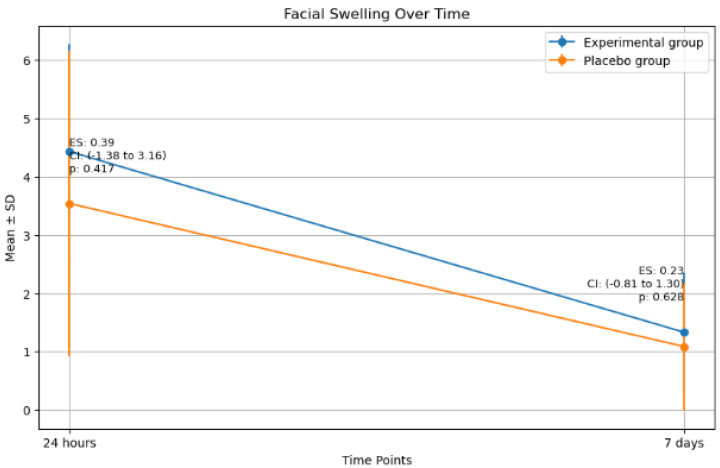
Mean facial swelling in the two groups 24 h and 7 days after the surgery. (ES: Effect size, CI: Confidence interval, *p*: *p*-value).

No significant differences in facial swelling were observed between the two groups either on the day after surgery or 7 days post-surgery (*p*-values = 0.417 and 0.628, effect sizes 0.4 and 0.2, respectively) (Figure 5).

A moderate, significant positive correlation was observed between the surgery duration and facial swelling at 24 h. Osteotomy also showed a moderate, significant correlation with facial swelling at 24 h. However, other variables did not exhibit any significant correlation with facial swelling at the different times assessed (Table 4).

### 2.5. Correlation Between Primary and Secondary Outcomes

After evaluating the correlation between the primary and secondary outcomes, a moderate, significant positive correlation was observed between pain at 12 h and facial swelling at 24 h, as well as between MMO change at 24 h and pain on the sixth day post-surgery. Additionally, pain from the second to sixth days post-surgery showed a moderate, significant positive correlation with MMO change at 7 days. Similar correlations were found between facial swelling at 24 h and MMO changes at both 24 h and 7 days, as well as between facial swelling at 7 days and MMO changes at 7 days. In addition, a significant moderate positive correlation was observed between pain from the second to the fifth day after surgery and PGE2 levels at three time points, indicating more pain reported in participants with a higher concentration of PGE2 in their saliva (Table 4).

### 2.6. Study Power Calculation

A post hoc power analysis utilizing an effect size of −0.63 in salivary PGE2 concentration after 24 h, a pooled standard deviation of 457.34, a sample size of nine, and a significance level of 0.05 revealed a study power of 20.43% in the laboratory part.

## 3. Discussion

This double-blind, randomized, parallel two-armed clinical trial demonstrated that administering 2 g of pre-operative amoxicillin had no significant impact on either clinical outcomes or salivary PGE2 concentration following lower third molar surgery. Correlation analyses indicated that a greater number of sutures were associated with increased pain on the day of surgery, while higher pain levels 6 days post-surgery were linked to a higher DI of the procedure. Additionally, a higher DI, longer surgery duration, and the implementation of osteotomy were found to contribute to more pronounced MMO changes. Conversely, extended surgery time and the performance of osteotomy were correlated with increased facial swelling. The results indicated that higher levels of pain were correlated with MMO changes, and MMO changes were correlated with facial swelling.

These results are consistent with previous studies indicating that pre-operative amoxicillin, with or without clavulanic acid, does not significantly impact postoperative clinical outcomes such as patient convenience, mouth opening, and pain [15,22]. However, another study found that prophylactic antibiotics reduced the need for painkillers [23], which was not evaluated in this study. While other articles have shown that pre-operative antibiotics can reduce the risk of surgical site infections [11,16,24,25], this study observed only one infection, and the rate of site infection was not significantly different between the two groups. In contrast to these findings, Torof et al.’s meta-analysis revealed a 69% reduction in infection risk in groups treated with antibiotics before or after surgery. However, similar to this study, their results indicated that different antibiotic regimens did not affect intraoral inflammation, wound dehiscence, lymphadenopathy, postoperative facial edema, trismus, or postoperative pain [14]. Despite the fact that meta-analyses and systematic reviews are more valuable than individual clinical trials, a recent evaluation of reviews revealed methodological issues in these systematic reviews, indicating that overall confidence in their results was low to very low [16]. Furthermore, the advantages of giving antibiotics to prevent infections in healthy patients having third molar surgery have been called into question by recent clinical trials [22,23,26]. Moreover, many published systematic reviews and meta-analyses also noted that many patients needed to be treated in order to observe the effects of antibiotic therapy [13,14,27,28].

Administering prophylactic antibiotics or pre-operative antibiotics can ensure high concentrations of antibiotics in the bloodstream and adequate levels at the surgical site before, during, and shortly after the procedure. This approach reduces the risk of bacterial invasions by common oral surgical infection pathogens such as Staphylococcus spp., Streptococcus spp., and anaerobic Gram-positive and Gram-negative rods [14,29]. Oppelaar et al. reviewed the efficacy of short-course (≤24 h) versus extended-course (≥72 h) antibiotic prophylaxis and found no significant difference in infection risk [30]. They recommended short-term antibiotics for ear, nose, throat, and oral and maxillofacial surgeries to minimize side effects like antibiotic resistance and disruption of the healthy microbiome, which can increase the risk of future infections and complications [20,30,31,32,33]. Additionally, one meta-analysis indicated that pre-surgical antibiotic administration for lower third molar extractions, particularly with osteotomy, is as effective as both pre- and postoperative prophylaxis in preventing surgical wound infections [21]. A systematic review also demonstrated that a single pre-operative dose of 2 g of amoxicillin significantly reduces the risk of surgical site infections [24]. Based on this background, we chose pre-operative antibiotic therapy to assess its impact on clinical outcomes following surgery.

Although there has been a declining trend in antibiotic prescriptions in dentistry in some countries [17], questionnaire-based studies have revealed that many dentists tend to prescribe antibiotics for all third molar surgeries. This practice aims to reassure patients and stems from the fear of potential post-surgery infections [19,34], often without considering the underlying causes or alternative preventive methods. Dentists play a crucial role in minimizing infection risks by ensuring good accessibility and visibility, and employing correct surgical techniques and instruments, guided by high-quality radiographs and an accurate assessment of the surgery’s difficulty [19,35]. Additionally, the use of Chlorhexidine mouthwash or gels to reduce the oral bacterial load before surgery is an effective strategy to mitigate post-surgery complications [36,37].

Furthermore, studies have emphasized that the surgeon’s experience, the duration of the surgery, and the extent of tissue trauma during extraction are critical factors influencing post-surgical complications [14,38]. To mitigate these variables, all surgeries in this study were conducted by an experienced oral surgeon. Despite the significant difference in DI between the two groups, the surgery duration was comparable. However, similar to previous findings, the results of this investigation revealed a correlation between the duration of surgery and acute postoperative symptoms such as facial swelling and changes in MMO [39]. Additionally, performing an osteotomy can lead to more long-term pain and short-term facial swelling and MMO changes, which have been identified as significant factors in postoperative complications in published studies [21,24].

Surgical removal of third molars induces a mild systemic inflammatory response that reflects in saliva, with the highest level of PGE2 after 24 h [3,40]. This study selected 24 h and 7-day post-surgery follow-ups to evaluate salivary PGE2 levels based on the findings of previous studies. Research has shown that the removal of impacted or semi-impacted teeth is associated with elevated inflammatory responses [5]. Factors such as impaction level, periodontal disease, and pre-existing infections contribute to increased inflammatory complications following third molar removal [41]. To minimize these effects, sound impacted third molars with healthy periodontal status entered the study. The results indicated that the DI of the surgery did not correlate with PGE2 concentration. Interestingly, longer surgery duration was associated with lower PGE2 concentrations in saliva at 7 days post-surgery, suggesting a quicker return to normal levels. This finding aligns with previous studies, which also found no correlation between the DI of the surgery and levels of salivary amylase or blood inflammatory factors [42,43]. However, this study found that performing tooth sectioning during surgery was correlated with lower PGE2 concentrations 24 h post-surgery. This may be due to the reduced pressure applied to remove the tooth from the socket.

PGE2 serves as a measurable post-traumatic marker for evaluating pain, fever, and inflammation [4]. The findings of this study confirmed this, showing that PGE2 concentrations correlated with reported pain from 48 h to 5 days after third molar surgery. Patients with higher baseline concentrations of PGE2 experienced more pain. However, Chandini R. et al. found no correlation between another salivary inflammatory factor, α-amylase, and reported pain after surgery. They evaluated saliva immediately after surgery, which is too soon to measure the inflammatory factors that cause pain [7]. Although it is challenging to measure PGE2 accurately in the blood, the scientific community recommends saliva as a well-accepted sample for its measurement [4]. This double-blind randomized clinical trial showed that antibiotic therapy administered one hour before mandibular third molar extraction surgery does not alter the inflammatory parameter in saliva. These findings are unique because, to the best of our knowledge, no prior study has examined the impact of preventive antibiotic medication on salivary PGE2 levels, and most publications have solely examined the clinical features of inflammatory sequela following third molar operations.

The primary limitation of this study was its low study power, which resulted from the small sample size. It is important to note that the small sample size (n = 9 per group) may have influenced the effect size estimates. Small sample sizes can lead to reduced statistical power, increasing the likelihood of Type II errors, where true differences or effects are not detected. Additionally, the variability in studied variables may be higher in small groups, leading to less precise estimates of the effect size. This increased variability can obscure true differences between groups and make the results less reliable. Therefore, the findings should be interpreted with caution, and further studies with larger sample sizes are recommended to validate these results. The stringent inclusion and exclusion criteria necessary for the laboratory component, along with many potential participants’ reluctance to forgo antibiotics or anti-inflammatory medications post-surgery, relying solely on analgesics for pain management, proved challenging and was another limitation of the study which lengthened the candidate selection process. Many potential participants believed that painkillers alone would be insufficient for managing their pain and inflammation. This underscores the need for more extensive community education about the adverse effects of long-term and unreasonable use of antibiotics and anti-inflammatories.

## 4. Materials and Methods

This double-blind, randomized, parallel two-armed clinical trial was conducted at the dental setting of Universidad Católica San Antonio de Murcia (UCAM) during 2023–2024. The study was approved by the UCAM ethical committee (CE032315) and submitted in clinicaltrials.gov (TRN: NCT06613776) and followed the CONSORT guideline.

The trial included adult patients (more than 18 years old) requiring at least one impacted mandibular third molar extraction according to the inclusion and exclusion criteria provided in Table 5. In line with the Declaration of Helsinki, researchers explained the study details and informed consent to the patients, including potential side effects and risks. Patient data were protected, and they could withdraw from the study at any time.

The sample size for the study was calculated using the results of the Stošić et al. [44] study in the formula (n = [(Zα/2 + Zβ)^2^ × (2(ó)^2^)]/(μ1 − μ2)^2^) with α = 5%, β = 10%, and a 30% dropout rate resulting in nine participants per group.

Eighteen patients were randomly assigned to two groups with a 1:1 allocation ratio. The EG received four capsules of 500 mg amoxicillin (Laboratorios NORMON, S.A., Madrid, Spain), totaling 2 g, one hour before surgery. The PG took four placebo capsules (empty gelatin capsules resembling amoxicillin), one hour before surgery.

All randomization and blinding processes were conducted by a third person who was not involved in any other steps of the study. Initially, the experimental and control groups were randomly designated as one and two. A computer-generated random set of twenty numbers (ten ones and ten twos) was created using www.calculatorsoup.com. The same person filled each of the twenty sealed packs with four capsules according to the random sequence, labeling them from 1 to 20. Patients received these labeled sealed packs sequentially, based on their surgery dates. The details of the packs remained unknown to the investigator, patients, surgeon, and statistical analyst.

**Figure 6:**
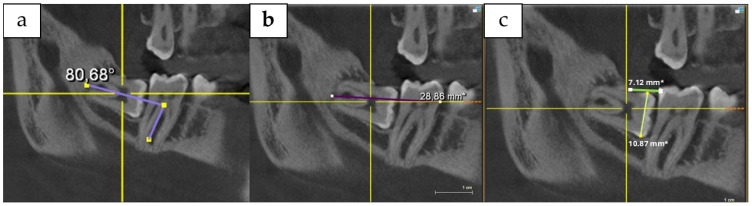
The difficulty index of the surgery was calculated by evaluating (**a**) spatial relationship, (**b**) depth, and (**c**) ramus relationship/space of third molar. In this method, the DI of the lower third molar extraction is measured using CBCT, based on its spatial relationship, depth, and ramus relationship/space. Each tooth is graded in each aspect, and the sum of these grades places the tooth into one of four categories: DI I to DI IV.

Before the surgery, each patient underwent Cone Beam Computed Tomography (CBCT) to evaluate the DI for surgical removal, following the method described by Jeong-Kui Ku et al. (Figure 6a–c) [35]. Additionally, basic periodontal examination (BPE) and gingival biotype assessment were used to evaluate the periodontal status of the patients [45,46].

**Figure 7:**
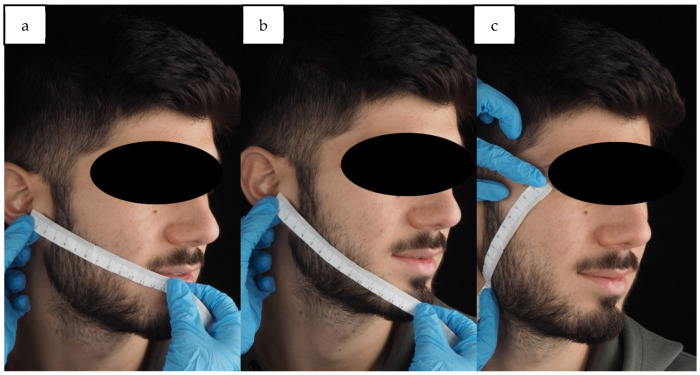
A paper ruler was used to measure facial dimensions: (**a**) the tragus distance to the corner of the mouth as line (**a**,**b**), the tragus distance to soft tissue pogonion as line (**b**,**c**), the distance of the outer corner of the eye to the angle of the mandible as line (**c**). One investigator measured facial dimensions using a tape measure while the participant sat upright at a 90° angle with their mandible at rest, to calculate the percentage of facial swelling using the formula ([postoperative measurement − preoperative measurement]/preoperative measurement) × 100.

One person used a paper ruler to measure facial dimensions, to calculate the facial swelling percentage after 24 h and 7 days, while participants sat upright at a 90° angle with their mandible at rest using the tape measuring method (Figure 7a–c) [47]. Additionally, MMO was evaluated by the researcher before surgery, immediately after surgery, and at 24 h and 7 days post-surgery. This was done by measuring the maximum distance between the incisal edges of the maxillary and mandibular incisors (in mm) using a metal ruler. The difference between the initial MMO and subsequent measurements was used for analysis.

To measure PGE2 concentration, 5 mL of unstimulated saliva were collected from patients at three different times: before taking the capsules, 24 h post-surgery, and seven days post-surgery. Patients refrained from eating or drinking for one hour before collection. They allowed saliva to flow into a cold, sterile tube without spitting. One milliliter of each sample was transferred into Eppendorf tubes to avoid multiple freeze–thaw cycles. These tubes, labeled with patient and session numbers, were stored at −80 °C until enzyme-linked immunosorbent assay (ELISA) analysis.

All surgeries were performed by a single surgeon under local anesthesia using an inferior alveolar block injection. A clean, full-thickness incision was made, followed by osteotomy and/or tooth section with abundant irrigation using 0.9% saline solution, if necessary.

Details of each surgery, including osteotomy, tooth sectioning, number of sutures, and the duration of the procedure (timed from the start of the incision to the removal of the last tooth fragment), were meticulously recorded.

The researcher provided patients with detailed postoperative instructions and a pamphlet outlining routine post-surgery care and permitted analgesics (Paracetamol 650 mg or Zaldiar 37.5 mg/325 mg, every 8 h for 7 days). Patients were instructed not to take any other medications, particularly antibiotics and anti-inflammatories, for one week after surgery. Additionally, they were asked to record their pain levels immediately after surgery; at 6 h, 12 h, and 24 h post-surgery; and daily at 17:00 for six days, using a Visual Analogue Scale (VAS) and 11-point pain scale.

During follow-ups 24 h and 7 days post-surgery, the investigator recorded secondary outcomes, including infection at the surgery site, dry socket, MMO, and facial measurements. In the first follow-up session, based on the patient’s reported pain and observed facial swelling, the surgeon prescribed Augmentin 875 mg/125 mg (every 8 h for 7 days) to ensure patients’ safety.

**Figure 8:**
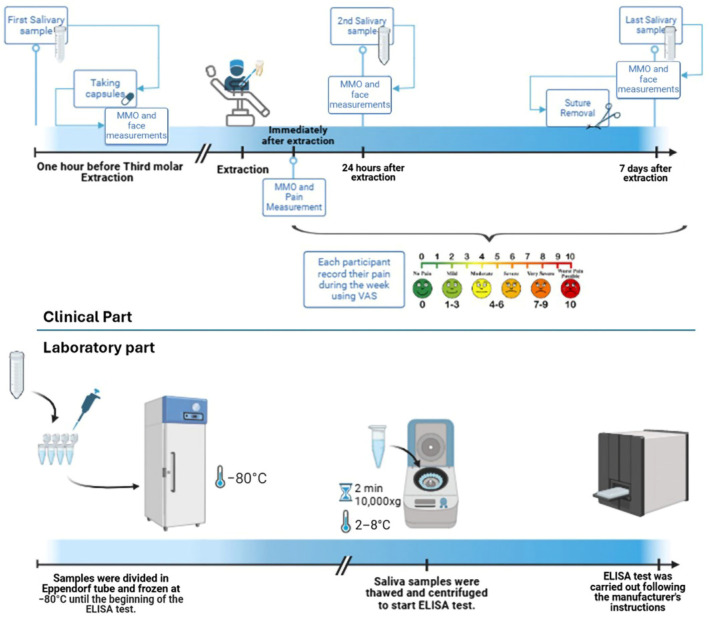
Summarized method of study. (Created in https://BioRender.com).

The concentration of PGE2 in saliva samples was measured using a PGE2 ELISA kit (Human PGE2 ELISA kit, EH4233, FineTest^®^, Wuhan Fine Biotech Co., Wuhan, China) employing the double-antibody sandwich technique. Saliva samples were rapidly thawed in a water bath at 20 °C to initiate the ELISA process. The samples were then centrifuged for two minutes at 10,000× *g* at 2–8 °C. The supernatant was collected and immediately used for the assay, following the manufacturer’s instructions. Optical density (OD) measurements for each well in the sample plates were taken to calculate the PGE2 concentration. Each specimen was tested duplicate to minimize laboratory errors, and the average value was calculated. To address the issue of extreme outliers in the PGE2 concentration data, the Winsorizing process was applied. Specifically, values below the 5th percentile and above the 95th percentile in each group at each time point were replaced with the values at the 5th and 95th percentiles, respectively. The exact values used for Winsorizing were as follows: for EG, PGE2 base values below 9.98 pg/mL were capped at 9.98 pg/mL, and values above 1378 pg/mL were capped at 1378 pg/mL; PGE2 after 24 h values below 46.1 pg/mL were capped at 46.1 pg/mL, and values above 1329 pg/mL were capped at 1329 pg/mL; PGE2 after 7 days values below 24.5 pg/mL were capped at 24.5 pg/mL, and values above 1157 pg/mL were capped at 1157 pg/mL. For PG, PGE2 base values below 42.1 pg/mL were capped at 42.1 pg/mL, and values above 1298 pg/mL were capped at 1298 pg/mL; PGE2 after 24 h values below 70.5 pg/mL were capped at 70.5 pg/mL, and values above 1367 pg/mL were capped at 1367 pg/mL; PGE2 after 7 days values below 8.24 pg/mL were capped at 8.24 pg/mL, and values above 1314 pg/mL were capped at 1314 pg/mL. The Winsorized data were then used for all subsequent statistical analyses. The method is summarized in Figure 8.

All data were entered into IBM SPSS Statistics (version 26.0; IBM, Armonk, NY, USA) according to group assignments and patient numbers. The samples were checked for normality using the Shapiro–Wilk Test. The Chi-square and Independent Samples *t*-tests were used to evaluate the baseline data similarity between two groups. The primary outcomes (patient-reported pain at various time points and salivary PGE2 concentration) and secondary outcomes (MMO change and facial swelling) were compared between the two groups using the Friedman, repeated measures ANOVA, Independent Samples t and Mann–Whitney U tests. Point-biserial correlation, Pearson correlation coefficient, and Spearman’s rank-order correlation were employed to assess the relationships between age, gender, surgery duration, osteotomy, tooth section, DI, number of sutures, and primary or secondary outcomes. A *p*-value of less than 0.05 was considered statistically significant. After completing the data analysis, the third person revealed the group assignments.

## 5. Conclusions

In conclusion, this study demonstrated that administering 2 g of prophylactic amoxicillin did not affect the incidence of infection, pain, salivary PGE2 concentration, MMO change, or facial swelling within one week of mandibular third molar surgery in healthy patients without pre-existing local infections at the surgical site. These findings highlight the need to carefully consider the use of antibiotics in dental surgery to prevent unnecessary prescriptions and combat antibiotic resistance.

## Figures and Tables

**Table 1:** Summary of the basic and surgery data of the Experimental group and Placebo Group.

		Experimental Group	Placebo Group	Total Number of Included Data	*p*
Gender, Number	Male	5	8	13	0.1 *
	Female	4	1	5	
Age (year), Mean ± SD		24.1 ± 4.4	25.7 ± 3.9	18	0.1 ^#^
Difficulty index (DI), Number	I	8	3	11	0.02 *
	II	1	6	7	
	III & IV	0	0	0	
Surgery duration (in seconds), Mean ± SD		1605.3 ± 1478.6	1205.1 ± 964.1	18	0.06 ^$^
Osteotomy, Number	Yes	5	5	10	1 *
	No	4	4	8	
Tooth section, Number	Yes	6	3	9	0.6 *
	No	7	2	9	
Number of tooth sections, Mean ± SD		3.2 ± 2.9	2.72 ± 3	9	0.4 ^#^
Number of sutures, Mean ± SD		3.4 ± 1.01	3.1 ± 0.9	18	0.2 ^#^

* Chi-square test, ^#^ Mann–Whitney U test, ^$^ Independent Samples Test, *p*: *p*-value.

**Table 2:** Pain, salivary PGE2 concentration, facial swelling, and MMO changes in the two groups at different time points.

		Group	N	Mean ± SD	Effect Size	*p*-Value
**Pain (Median ± IQR)**	**After surgery**	Experimental	9	1 ± 3	0.3	0.2
		Placebo	9	0 ± 1.5		
	**6 h**	Experimental	9	6 ± 2	0.3	0.2
		Placebo	9	4 ± 5		
	**12 h**	Experimental	9	5 ± 3	0.01	0.9
		Placebo	9	4 ± 7.5		
	**24 h**	Experimental	9	5 ± 1.5	0.2	0.4
		Placebo	9	3 ± 8.5		
	**48 h**	Experimental	9	4 ± 3.5	0.0	1.0
		Placebo	9	2 ± 7.5		
	**72 h**	Experimental	9	2 ± 4.5	0.05	0.8
		Placebo	9	2 ± 7		
	**4th day**	Experimental	9	1 ± 4	0.1	0.6
		Placebo	9	4 ± 5.5		
	**5th day**	Experimental	9	1 ± 3	0.3	0.2
		Placebo	9	2 ± 3		
	**6th day**	Experimental	9	1 ± 3	0.3	0.2
		Placebo	9	1 ± 3.5		
**Salivary PGE2 concentration (pg/mL)**	**Baseline**	Experimental	9	422.5 ± 436.8	−0.6	0.2
		Placebo	9	687.9 ± 497.0		
	**24 h**	Experimental	9	487.8 ± 453.3	−0.6	0.2
		Placebo	9	760.3 ± 461.3		
	**7 days**	Experimental	9	652.3 ± 513.7	−0.01	0.9
		Placebo	9	657.6 ± 445.8		
**Facial swelling (%)**	**24 h**	Experimental	9	4.4 ± 1.8	0.4	0.4
		Placebo	9	3.5 ± 2.6		
	**7 days**	Experimental	9	1.3 ± 1.0	0.2	0.6
		Placebo	9	1.1 ± 1.1		
**MMO changes (mm)**	**After surgery**	Experimental	8	4.1 ± 3.4	−0.03	0.7
		Placebo	9	4.2 ± 2.1		
	**24 h**	Experimental	9	17.9 ± 12.6	−0.06	0.9
		Placebo	9	18.56 ± 11.6		
	**7 days**	Experimental	9	13.0 ± 11.0	0.3	0.5
		Placebo	9	9.4 ± 9.5		

**Table 3:** Correlation between pain and other variables and secondary outcomes. Positive Correlation Coefficient (CC) means that reported pain raised by increasing the variable.

		Pain								
		After	6 h	12 h	24 h	48 h	72 h	4 D	5 D	6 D
**Gender**	CC	−0.1	−0.3	−0.3	−0.4	−0.5 *	−0.4	−0.3	−0.1	−0.05
	*p*	0.7	0.2	0.16	0.13	0.03	0.07	0.2	0.6	0.8
**Age**	CC	−0.6 *	0.1	−0.06	−0.03	−0.04	−0.17	−0.2	−0.2	−0.3
	*p*	0.01	0.6	0.8	0.9	0.9	0.5	0.3	0.5	0.2
**DI**	CC	0.0	−0.3	0.2	0.02	0.0	0.2	0.3	0.4	0.6 *
	*p*	1.0	0.3	0.5	0.9	1.0	0.4	0.3	0.08	0.01
**Tooth section**	CC	0.0	−0.04	0.01	−0.02	−0.17	−0.2	−0.1	−0.1	−0.05
	*p*	1.0	0.9	0.9	0.9	0.5	0.4	0.7	0.6	0.8
**Number of tooth sections**	CC	0.2	−0.06	−0.06	−0.05	−0.4	−0.3	−0.3	−0.35	−0.09
	*p*	0.4	0.8	0.8	0.8	0.14	0.18	0.3	0.1	0.7
**Number of sutures**	CC	0.1	0.5 *	0.6 *	0.3	0.4	0.2	0.1	0.04	0.12
	*p*	0.6	0.01	0.01	0.2	0.1	0.4	0.6	0.9	0.6
**Surgery duration (S)**	CC	0.2	−0.1	0.05	−0.01	−0.2	−0.16	−0.1	−0.05	0.3
	*p*	0.4	0.7	0.8	0.9	0.3	0.5	0.6	0.8	0.3
**Osteotomy**	CC	0.05	−0.02	0.2	0.2	0.2	0.3	0.2	0.4	0.6 **
	*p*	0.8	0.9	0.4	0.4	0.5	0.3	0.4	0.1	0.007

** Correlation is significant at the 0.01 level. * Correlation is significant at the 0.05 level. CC: Coefficient Correlation.

**Table 4:** Correlation assessment of salivary PGE2 concentration and clinical outcomes with other variables and together.

			PGE2 Concentration			MMO Changes			Facial Swelling	
			Baseline	24 h	7 D	After	24 h	7 D	24 h	7 D
Gender		PC	0.2	−0.2	−0.2	0.4	0.08	−0.2	−0.3	−0.3
		*p*	0.5	0.5	0.3	0.15	0.7	0.3	0.2	0.2
Age		PC	0.15	−0.2	−0.2	−0.1	−0.45	−0.3	−0.4	−0.3
		*p*	0.5	0.3	0.3	0.7	0.06	0.2	0.07	0.3
DI		CC	0.08	0.03	−0.15	0.3	0.63 **	0.3	0.2	0.2
		*p*	0.8	0.9	0.6	0.3	0.005	0.2	0.4	0.4
Surgery duration		PC	−0.3	−0.4	−0.5 *	0.67 **	0.5 *	0.4	0.5 *	0.1
		*p*	0.2	0.06	0.03	0.003	0.02	0.15	0.03	0.6
Osteotomy		PC	−0.004	−0.1	−0.2	0.5	0.8 **	0.68 **	0.5 *	0.07
		*p*	0.9	0.6	0.4	0.06	0.0	0.002	0.03	0.8
Tooth section		PC	−0.05	−0.5 *	−0.3	0.2	0.2	0.1	0.1	−0.02
		*p*	0.8	0.04	0.2	0.4	0.4	0.6	0.6	0.9
Number of tooth sections		PC	0.3	0.16	0.25	0.2	0.3	0.1	0.4	0.1
		*p*	0.2	0.5	0.3	0.4	0.2	0.6	0.1	0.5
Number of sutures		PC	0.3	0.16	0.25	0.2	0.3	0.4	0.3	0.2
		*p*	0.2	0.5	0.3	0.3	0.2	0.1	0.3	0.3
Facial Swelling	24 h	PC	−0.15	−0.07	−0.07	0.5	0.66 **	0.6 **	1	0.6 **
		*p*	0.5	0.8	0.8	0.05	0.003	0.005		0.007
	7 D	PC	0.25	0.07	0.2	−0.004	0.4	0.51 *	0.6 **	1
		*p*	0.3	0.8	0.4	0.9	0.1	0.03	0.007	
MMO changes	After	PC	−0.1	−0.16	−0.2	1	0.62 **	0.4	0.5	−0.004
		*p*	0.6	0.5	0.3		0.008	0.1	0.05	0.9
	24 h	PC	0.2	−0.02	0.1	0.62 **	1	0.85 **	0.66 **	0.4
		*p*	0.4	0.9	0.6	0.008		0.0	0.003	0.1
	7 D	PC	0.3	−0.03	0.2	0.4	0.85 **	1	0.6 **	0.51 *
		*p*	0.2	0.9	0.3	0.1	0.0		0.005	0.03
Pain	After	CC	−0.1	−0.03	0.03	0.3	0.09	0.1	0.4	0.4
		*p*	0.7	0.9	0.9	0.2	0.7	0.6	0.07	0.1
	6 h	CC	0.4	0.2	0.3	0.07	0.06	0.3	0.4	0.3
		*p*	0.07	0.4	0.2	0.8	0.8	0.2	0.1	0.2
	12 h	CC	0.3	0.4	0.2	0.13	0.4	0.4	0.5 *	0.3
		*p*	0.2	0.07	0.5	0.6	0.1	0.07	0.02	0.3
	24 h	CC	0.3	0.3	0.25	−0.06	0.2	0.4	0.4	0.2
		*p*	0.2	0.2	0.3	0.8	0.5	0.08	0.08	0.3
	48 h	CC	0.5 *	0.5 *	0.5 *	−0.06	0.2	0.5 *	0.3	0.4
		*p*	0.04	0.03	0.03	0.8	0.4	0.02	0.2	0.1
	72 h	CC	0.5 *	0.5 *	0.5 *	0.04	0.3	0.6 **	0.35	0.4
		*p*	0.04	0.04	0.02	0.9	0.2	0.008	0.15	0.1
	4 D	CC	0.6 *	0.6 *	0.6 **	0.08	0.3	0.6 **	0.3	0.3
		*p*	0.01	0.01	0.005	0.7	0.2	0.007	0.2	0.2
	5 D	CC	0.5 *	0.5 *	0.5 *	0.3	0.4	0.6 **	0.3	0.04
		*p*	0.03	0.01	0.04	0.3	0.08	0.009	0.3	0.9
	6 D	CC	0.1	0.3	0.05	0.3	0.5 *	0.5 *	0.4	−0.01
		*p*	0.7	0.2	0.8	0.2	0.02	0.02	0.1	0.9

** Correlation is significant at the 0.01 level. * Correlation is significant at the 0.05 level. PC: Pearson Correlation, CC: Coefficient Correlation.

**Table 5:** Inclusion and exclusion criteria of the patients included in the study.

Criteria	Details
Inclusion Criteria	Adult patients (more than 18 years old)
	Requiring at least one impacted mandibular third molar extraction
	Healthy (ASA I and II)
	Non-smokers
	No history of viral or microbial diseases in the past four months
	No known allergies to penicillin-class antibiotics or other drugs
	No use of anti-inflammatory or contraceptive drugs in the past month
	No autoimmune diseases
	Not pregnant or nursing (for women)
Exclusion Criteria	History of dental pain, inflammation, or abscess in the past month
	Thyroid hormone therapy
	Unwillingness to participate in the study

## Data Availability

The original contributions presented in the study are included in the article; further inquiries can be directed to the corresponding author.

## Abbreviations

The following abbreviations are used in this manuscript:

PGE2	Prostaglandin E2
PG	Placebo Group
EG	Experimental Group
	Standard Deviation
DI	Difficulty Index
MMO	Maximum Mouth Opening
CC	Correlation Coefficient
PC	Pearson Correlation
